# How calling emerges and develops during COVID-19: a qualitative study of medical students

**DOI:** 10.1186/s12909-023-04914-w

**Published:** 2023-12-08

**Authors:** Jia Xu, Baoguo Xie, Tingting Liu, Jie Li

**Affiliations:** 1 Department of Psychology, Wuhan University, Wuhan, 430072 China; 2 School of Management, Wuhan University of Technology, Wuhan, 430070 China; 3 Department of Management, Shanghai University of International Business and Economics, Shanghai, China; 4 Department of Strategy and Organization Management, Xi’an Jiaotong-Liverpool University, Suzhou, China

**Keywords:** COVID-19, Calling, Emergence, Medical students

## Abstract

**Introduction:**

The presence of calling in medicine has been shown to be related to a preponderance of positive outcomes among medical students. However, only a few studies examined the antecedents of calling. Of this group, little is known about how a calling emerges and develops in a crisis situation. This study examines the processes underlying the emergence and development of calling when confronted with COVID-19.

**Methods:**

Semi-structured interviews were conducted with medical students (*N* = 28) from China from February to March 2020. Medical students reported their experiences about the emergence of calling, its antecedents, and its outcomes in the context of the COVID-19 pandemic. Interviews were transcribed and analyzed using a thematic analysis approach.

**Results:**

Four main themes were identified: (1) the definition of calling, (2) the trajectories of calling development, (3) the factors leading to the emergence of calling, and (4) the outcomes of the emergence of calling. Medical students conceptualized calling as both self- and other-oriented regarding serving the common good. Three calling paths were revealed: significantly enhanced, growing out of nothing, and remaining unchanged. Work sense-making and identity formation interact to facilitate the emergence of calling. The emergence of a calling affects career and study-related outcomes.

**Discussion:**

Our findings advance the concept of how the calling of medical students emerges and develops in response to life events through work sense-making and identity formation. Academic institutions and medical educators could leverage these events to facilitate calling discernment among medical students.

## Introduction

Occupational calling as a multidimensional construct that describes motivational, affective facets of the relationship between individuals and work domains [1, 2], has gained much attention from scholars over the past 10 years [3]. Students identifying with medicine as a calling are more likely to experience increasing well-being [4], specialty commitment, self-efficacy [5], and active engagement in vocational development tasks [4, 6]. Given the role of calling in medical students’ careers and lives, it is essential to understand the process through which a calling emerges and develops [7].

The emergence of a calling has been linked to various factors, such as certain personal characteristics [8], familiarity and comfort with the calling domain, behavioral involvement [9], and interaction with the key influencers in the calling domain [8, 10]. However, more research is needed to explore how a calling emerges and is discovered [11, 12]. Furthermore, previous research examining the specific life experiences shaping one’s calling failed to clarify the process of discerning a calling [13, 14]. This is particular important because people may scrutinize their career choices after negative life events [15, 16]. Finally, the diverse conceptualizations of calling have caused confusion [12, 17]. From the classical or neoclassical viewpoint, calling can be conceptualized as destiny or prosocial duty, and in the modern or secular view, calling emphasizes passion and self-fulfillment [17]. A need exists to bridge these two poles of the continuum. Exploring the definitions of calling among medical students may clarify differences in scholars’ views on the core features of a calling.

As a public event, coronavirus disease 2019 (COVID-19) have disrupted people’s understanding of the world and changed their lives in different ways [18]. Generally, when faced with critical incidents or life events such as the COVID-19 pandemic, people are prompted to discover and rebuild meaning in life [15], and identify and pursue a calling [1]. For example, Turkish nurses experienced changes to their sense of calling during the COVID-19 pandemic [19]. Thus, COVID-19 provides a relevant context for exploring the open questions of how calling is defined and emerges among medical students.

By March 8, 2020, more than 42,600 medical staff and 1,800 epidemiological teams in China had participated in treating COVID-19 patients [20]. The career experiences of medical staff may have disproportionate effects on medical students. Hence, interviews with medical students could provide rich data for our research on how COVID-19 shaped medical students’ calling experiences.

## Methods

### Participants

Participants were selected from a medical university in southwestern China, from which five medical aid teams had participated in the direct treatment of patients during the outbreak of COVID-19 in China. Participants were recruited by offline (convenience sampling and referral sampling) and online (websites and WeChat) methods. We contacted them by email, asked for their demographic information for reference, and ultimately selected 28 participants for in-depth interviews.

The 28 medical students for the in-depth interview were not involved in direct patient-care activities due to the students’ protection policy; however, they actively participated in anti-epidemic volunteer activities, such as distributing supplies and community services. Most of them majored in clinical medicine, while others majored in preventative medicine, anesthesia, traditional Chinese medicine, stomatology, and nursing. The 28 medical students (aged 19–23) were in different grades; ten were male and 18 females. The respondents participated voluntarily and signed an electronic consent for the interview. The interviewees are described in Table 1, with pseudonyms used to protect their identities.

**Table 1:** List of study’s participants for interview

*No*	*Name*	*Age*	*Gender*	*Grade*	*Major*
1	Mu	23	Woman	Senior	Clinical medicine
2	Fang	20	Woman	Sophomore	Traditional Chinese medicine
3	Ai	19	Woman	Freshman	Clinical medicine
4	Song	20	Woman	Junior	Clinical medicine
5	Qian	20	Woman	Sophomore	Preventive medicine
6	Wang	20	Man	Sophomore	Clinical medicine
7	Tan	20	Woman	Sophomore	Clinical medicine
8	Zhao	20	Man	Sophomore	Clinical medicine
9	Xia	19	Man	Sophomore	Clinical medicine
10	Zeng	21	Man	Junior	Preventive medicine
11	Yao	22	Man	Senior	Clinical medicine
12	Zhong	20	Man	Sophomore	Anesthesia
13	Yi	20	Woman	Sophomore	Clinical medicine
14	Wu	20	Woman	Sophomore	Clinical medicine
15	Qu	20	Woman	Sophomore	Clinical medicine
16	Guo	20	Woman	Sophomore	Stomatology
17	Wang	20	Man	Sophomore	Clinical medicine
18	Xu	20	Woman	Sophomore	Clinical medicine
19	Su	23	Woman	Senior	Anesthesia
20	Xia	21	Man	Sophomore	Clinical medicine
21	Hua	19	Woman	Freshman	Clinical medicine
22	Liu	20	Man	Sophomore	Clinical medicine
23	Gu	19	Woman	Sophomore	Nursing
24	Hua	21	Woman	Sophomore	Preventive medicine
25	Chen	20	Woman	Senior	Clinical medicine
26	Geng	19	Woman	Sophomore	Clinical medicine
27	Di	20	Woman	Sophomore	Clinical medicine
28	Liang	21	Man	Senior	Clinical medicine

We believe our participant selection is consistent with the aim of this study. Undergraduate medical students are at the initial stage of their career development, during which career exploration behaviors are abundant [8]. They are sensitive to external events related to their careers. Moreover, medical students’ professional identities and career choices are more easily affected by a series of anti-epidemic behaviors than those of healthcare professionals with specific working experience and a relatively stable professional identity [21]. Hence, this group provides rich data for our research on how COVID-19 shapes medical students’ calling experience.

### Data collection

Semi-structured interviews were conducted from February to March 2020 to collect data. The interview protocol was open-ended and exploratory. The interviews started with general inquiry questions. Answers to these questions are critical for understanding whether medical students have experienced changes in their study and life after COVID-19. The calling-specific questions were clustered to reflect the emergence of calling, its antecedents, and outcomes during COVID-19. The questions were as follows (in Chinese, translated here to English): (a) What is your definitions of calling in the context of COVID-19? (b) How do you describe the calling change during the COVID-19? (c) What are the experiences during COVID-19 that facilitates the development and understanding of a sense of calling? (d) Does the calling impact your future? Please explain.

We conducted phone and online video interviews due to social isolation during the COVID-19 outbreak. Each interview took approximately 60–90 min to complete. All interviews were tape-recorded and transcribed, and the memorandums were written within 24 h after the interviews. The audio recordings and field notes of the participants’ reflections served as additional data sources.

### Data analysis

We conducted data analysis based on the thematic analysis [22]. We tried to remain open to our respondents’ accounts and added emergent codes as we analyzed the transcripts. The core data analysis team comprised the first and second authors. We started data analysis by reading transcripts multiple times and writing memorandums to note any patterns emerging from the data. The data were then categorized and integrated using the most important or frequent initial codes. We maintained sensitivity during data analysis and constantly compared emerging ideas from the data to identify themes. We coded transcripts of more than 240,000 words and generated in vivo codes (the expressions and terms used by participants).

The coding process comprised two steps. The first step was reading all the transcripts independently to generate inductive codes. With coding, each word, sentence, paragraph, and passage is a unit of text. Codes are shorthand terms used to categorize units of text. The second step was comparing, discussing, and reflecting on the codes through regular joint coding meetings. The final codes were determined by consensus. During each meeting, the noncoding author served as a “judge” to read the passage in question, examine the texts, and suggest appropriate codes to help in the final code agreement. We revisited the interview transcripts multiple times when necessary to check the relevance and accuracy of codes and resolve areas of disagreement between the authors. Discussions also helped develop and refine the codes, and produced rich opportunities for refining and naming themes.

### Reflexivity

The research group comprised senior and junior researchers with rich research experience in the area of students’ career development. JX works at a university that cooperates extensively with hospitals and medical institutions and had access to much data during COVID-19. BGX provided theoretical insights into the calling study. TTL has extensive qualitative research experience and trained the team on conducting semi-structured interviews. JL has particularly strengthened the methodological rigor of the study. These varied backgrounds and experience in the author group allowed us to interpret the data from different angles.

## Results

Data from the interview were organized into four main themes: (1) the definition of calling, (2) the trajectories of calling development, (3) the factors leading to the emergence of calling, and (4) the outcomes of the emergence of calling. Table 2 provides an overview of these themes, their sub-themes and frequencies.

**Table 2:** List of theme, sub-theme and frequencies

Theme	*Sub-theme*	*n*	*%*
Definition of calling	Moral agent	28	100
	Professional responsibility	21	75
	Dedication to serving the common good	26	92.9
Trajectories of the Emergence of Calling	Significantly enhanced	17	60.7
	Growing out of nothing	7	25
	Remaining unchanged	4	14.3
Factors Leading to the Emergence of a Calling	Work sense-making	21	75
	Identity formation	23	82.1
Impacts of the Emergence of a Calling	Career decidedness	26	92.9
	Career preparation	9	32.1
	Intrinsic study motivation	28	100
	Study involvement	17	60.7

### Theme 1: Definition of calling

In explaining medical students’ definitions of a calling, three sub-themes emerged: *moral agent*, *professional responsibility*, and *dedication to serving the common good*.

#### Theme 1.1 Moral agent

All the interviews referred to calling as a *moral agent* reflecting the importance of the ethical dimensions of medical work. The participants perceived that medical staff should be able to make moral choices in the healing relationship and be responsible for furthering patients’ best interests. For example, one participant stated,

*“As we all mentioned, the medical staff are angels in white coats. I feel that since I have chosen this profession, I will be expected to meet the demands of morality and benefit or limit harm to patients.”*

#### Theme 1.2 Professional responsibility

More than half (75%) of the interviewees also typically viewed their calling as a *professional responsibility* constituting a normative behavior versus an individual choice. Individuals’ responses falling under this theme often expressed a sense of duty to provide medical services despite obstacles. For example, one participant stated,

*“I think most of us agree that doctors have a sense of responsibility. Since the outbreak of the epidemic, medical personnel have rushed to the battlefield from all corners of the country, not afraid of difficulties or death, to overcome the impact of the epidemic. This is a kind of constant professionalism and a sense of professional mission engraved in the bones.”*

#### Theme 1.3 Dedication to serving the common good

Additionally, the overwhelming majority (92.9%) of the interviewees viewed a calling as a *dedication to serving the common good*, reflecting how one gives one’s energy, skill, and commitment to working with patients, suggesting their interest and passion regarding calling are not merely for self-realization, but reflect a need to promote the common good. For example, one participant stated,

*“The calling of a doctor comes from two points: the first point is love, and the second point is to treat diseases and save people. And the sense of calling is more derived from love. Love is not only the love of medicine but also the love of the people around you. I am full of love for this profession and look forward to helping others, and I admire my classmates very much.”*

### Theme 2: Trajectories of the emergence of calling

The medical students described differences in their calling development. All participants in our study highlighted similar types of callings; they encountered similar challenges during COVID-19. However, they responded to COVID-19 in different ways. Based on these responses, we identified three calling paths: *significantly enhanced*, *growing out of nothing*, and *remaining unchanged*.

#### Theme 2.1: Significantly enhanced

“Significantly enhanced” describes how individuals perceived a significant enhancement in calling during COVID-19. The majority (60.7%) of participants identified increased calling. For example, one participant stated,

“At the time of admission, all medical students need to take the Hippocratic oath. At that time, I felt very moved, but I did not have much in-depth experience. In this epidemic, we saw the teachers and the medical staff working hard on the front line. I was really moved by their dedication; I have a better understanding of the mission and significance of the profession of medicine.”

**Theme 2.2: Growing out of nothing**

“Growing out of nothing” describes how individuals on this path responded to the COVID-19 pandemic and began to find or perceive their callings. Seven participants experienced growth out of nothing during the calling development process. One participant elaborated,

“At first, I was forced by my parents to choose this major, and then I didn’t really agree with it in my heart. However, this pandemic should be a great wake-up call for me, for our professionals, and people in this field. I think what I want to do is to use my professional ability to protect people’s health.”

#### Theme 2.3: Remaining unchanged

“Remaining unchanged” describes individuals who were called but did not perceive a significant change in calling perception before or during the COVID-19 pandemic. Four medical students noted their calling had not significantly changed. Among them, two participants were unaware of any changes in their calling. For example, one participant stated,

“In this epidemic, my perception of the meaning of this profession has not changed much. I really think it was the same before and after the pandemic. As a medical student, I can foresee what it will look like if there is a public health event. I thought about this before I chose the medical specialty. Therefore, from my point of view, there is no great change.”

Another two participants stated that they could not even feel a calling. One of the participants described this as follows,

*“To tell you the truth, I haven’t even stepped into the career path. I think it is a bit unrealistic to say that I’m looking for a sense of calling. I just can’t feel this feeling.”*

### Theme 3: Factors leading to the emergence of a calling

In this section, the medical students reported core factors to explore how two calling paths produced calling emergence and development, while the third calling path was associated with no overall change. Qualitative analysis revealed that most participants identified the emergence of a calling as characterized by an interplay between *work sense-making* and *identity formation*.

#### Theme 3.1: Work sense-making

Work sense-making was a key topic of discussion among most medical students when discussing what facilitates the emergence of calling. COVID-19 violated participants’ global beliefs about the world and themselves; such violations initiated sense-making to rebuild their meaning systems to make them less averse to negative effects and thus accommodate the experience [18]. Twenty-one participants indicated that they tended to make sense of medical work to adapt to the COVID-19 pandemic and reported two primary characteristics of work contributing to developing a calling: task significance and social worth.

##### Task significance

Participants with calling experience indicated task significance was a major factor influencing their emergence of calling; this describes the degree to which a job leads to improving the welfare of others. During education and training in the university, medical students struggle to understand the purpose of medical professions and often consider the medical profession as a tool to satisfy material needs. By observing career predecessors fighting the pandemic, medical students gained a broader perspective of the medical profession and realize they could make meaningful contributions to the common good. For example, one participant described how she perceived the importance of the medical occupation:

* “When I was in class before, I felt that those theories were not of great significance. I thought that the pandemic could only be seen in movies. But this time, the pandemic is really happening around us. Seeing the efforts of many teachers and senior students, I began to rethink the role of preventive medicine as a discipline. I deeply realized the importance of this occupation.”*

##### Social worth

Participants mentioned their perception of others’ increasing appreciation of medical staff for their actions for patients during the pandemic. In previous years, some doctors and nurses were attacked, injured, or killed by their patients or patients’ family members over disputes between medical personnel and patients in China [23]. However, the positive impact of medical work on society during COVID-19 made patients value efforts of medical staff. According to participants, this sense of being valued by others was an important factor contributing to their calling emergence and development. For example, one participant stated,

* “This epidemic has shown us the responsibility of medical staff. We can see that almost all medical staff are at great risk of infection. Then through various reports, the public can realize this. I think this epidemic can make more people cherish the contributions of medical staff and respect them. Thus, we witnessed that thousands of people lined up in the streets to give medical staff a warm send-off when they returned home.”*

#### Theme 3.2: Identity formation

The overwhelming majority (82.1%) of the participants also perceived their *professional identity formation* as related to calling emergence and development. Professional identity formation, as one of the main goals of a medical education program, involves a focus on who the students are becoming and what they know or can do [8]. We found that the professional identity formation is another trajectory of calling emergence and development, from which two routes have been identified: *clarity of professional identity* and *in-group identification*.

##### Clarity of professional identity

Participants perceived themselves as internalizing the characteristics, values, and norms of the medical professionals and identified strongly with medicine. They expressed a greater understanding of their attributes, beliefs, motives, and experiences through their definition of professional roles. For example, one participant stated,

“As a doctor, one of the most familiar phrases is ‘saving the dying and helping the wounded,’ but our experience was not very deep. After all, I just stepped over the threshold of medicine, and my understanding of the profession was at its most basic. However, in this epidemic, through news reports, I learned about many great medical staff and their deeds. Now I know that the most important thing is the sense of responsibility and mission.

##### In-group identification

Participants expressed their identification with the medical personnel group. They viewed themselves as (future) medical professionals, leading them to make more psychological and behavioral commitments to the medical group. For example, one participant stated,

* “Many of the teachers at our university went to the front line to fight against the epidemic. The epidemiology teacher gave online lessons to us in the command center while fighting against the epidemic. I feel immensely proud when others praise medical staff, because I am also a member of the medical profession. I have a deeper perception of the professional mission of medical workers. I hope that I can truly stand with them in the future.”*

#### Theme 4: Impacts of the emergence of a calling

In answering, “Does the calling impact your future? Please explain,” most of the participants mentioned that changes in perceiving a calling led to changes in career-related and study-related outcomes.

#### Theme 4.1: Career decidedness

Most (92.9%) participants affirmed their choice of occupation and career decision. They experienced increased confidence or certainty about their choices in their medical occupation. For example, one participant stated:

*“Influenced by my family, I was confused about choosing this major, and then I planned to go to any hospital closer to home after graduation. But now I feel that I really want to devote myself to the profession of medicine, and I want to research and treat diseases, because I am clearer and more determined about my future career path.”*

#### Theme 4.2: Career preparation

*Career Preparation* A few (32.1%) participants described career preparation as the outcome of calling emergence and development. They expressed their future direction and purpose and outlined a career plan and active strategies to achieve career goals. One participant elaborated,

*“This epidemic has had some impact on my career direction. I originally wanted to choose the surgical direction. Due to this epidemic, I now prefer internal medicine. In the future, I really want to experience the feeling of fighting on the front line. I think about going to graduate school in the future, doing varied research, writing professional papers, and preparing for the recurrence of similar epidemics. We must make more preparations to develop and enrich professional knowledge.”*

#### Theme 4.3: Intrinsic study motivation

All students who felt called found that their calling influenced their study motivation. They described their experience of school learning for its own sake, where pleasure is inherent in the learning process. One such participant described the following:

*“In this epidemic, my learning motivation is stronger. This crisis made me very alert, letting me realize that medical work especially needs professional knowledge. I used to think that as long as I passed the exam, it would be OK. But now I have found that only by gaining all the knowledge and skills and accumulating experience can we make contributions in this field and protect the health of others.”*

#### Theme 4.3: Study involvement

More than half (60.7%) of the participants suggested that a calling increased their willingness to exert effort at school. They intended to devote more physical and psychological energy to their academic activities. One participant stated:

*“Now, the first thing is to learn professional courses well, because I realize that this professional knowledge is especially useful in dealing with public health emergencies. We must study hard, try our best to improve the national emergency prevention system and try our best. When similar events happen in the future, we could be more fully prepared and more capable of dealing with these emergencies.”*

## Discussions

This study aimed to understand how medical students viewed the experiences leading them to their career callings. Our findings suggest that callings can be both self- and other-oriented regarding serving the common good. Furthermore, several factors shaping a calling after COVID-19 are identified. The findings show that work sense-making (i.e., task significance, and social worth) and identity formation (i.e., clarity of professional identity, and in-group identification) are two aspects that interact to facilitate the emergence of calling. Finally, the emergence of a calling affects career-related (i.e., career decidedness, and career preparation) and study-related outcomes (i.e., intrinsic study motivation, and study involvement).

Our study makes three contributions to the calling and coping literature. First, our research adds insights to the dialogue on what calling means by exploring the experiences of medical students. Scholarship is undecided on a definition of calling [12]. Our findings reveal that the participants perceived their calling as emphasizing other-oriented purpose, duty, and dedication as well as self-oriented interests and passion. Furthermore, the other- and self-oriented callings are not mutually exclusive, as participants were thought to be pursuing a personal interest or passion (serving the self) toward a public good (serving others). These findings resonate with Michaelson and Tosti-Kharas’s [24] point that “self-orientation can be consistent with cultivating an ethical life and is not incompatible with other orientation” (p. 4).

Second, this study clarifies the process of the emergence of calling. Despite acknowledging its association with life events, most studies have not developed a theoretical model explaining the mechanisms responsible for its existence [11, 14]. The present study found that two processes play a dominant role in calling emergence: work sense-making and identity formation processes. In the past, both Bunderson and Thompson [25] and Carton [26] have observed that calling is associated with the belief that one’s work contributes to the greater good. However, the researcher still does not know whether social worth induces individuals to develop career calling. As described above, when experiencing deep meaningfulness (e.g., significance and socially value) and identity formation (e.g., clarity of professional identity and in-group identification), participants will be more committed to their work and are called within the career path. Social identity theory also suggests that professional groups with higher status, prestige, salience, and competition are appealing [27], because these characteristics offer increased esteem enhancement [28]. Thus, our findings elucidate more detail than previous studies how calling emerges. Although recent research on calling has begun to consider both work characteristics and professional identity [26, 29], this study is the first to treat these factors as two parallel mechanisms for the emergence of calling.

Finally, our findings contribute to the literature concerning adaptation to adverse life events. Considerable interest exists in how individuals manage adverse life events [30, 31], but little is known about variations in how individuals appraise and cope with events and the extent to which these coping processes operate together to influence career outcomes. Existing literature suggests that individuals respond to adversity differently, including passive acceptance and positive coping [32, 33]. The COVID-19 pandemic is an adverse event and negatively associated with the psychological and physical well-being of medical staff [34]. Our results suggest that the “context” of calling in response to COVID-19 involves more than the usual exposure to (the threat of) infection and human suffering. It serves as a turning point and represents a time of upheaval during which the characteristics of work and professional identity are reexamined, through which medical students identify calling emergence and report increased motivation toward study and career development. These findings underscore the need to integrate calling emergence into the coping literature.

Knowledge of how calling emerges and develops during adversity such as COVID-19 has important implications for medical education. First, the current study provides enlightening knowledge of how to facilitate the callings of medical students. Since having a calling is associated with psychological and vocational benefits [4–6], educators must help medical students find and fulfill their callings. Our study raises the important role of work sense-making and a clear professional identity in the process of calling emergence. Educators should highlight how one’s work contributes to others and society and should not minimize the importance of “small” or “routine” work tasks during the internship to bolster calling emergence. Meanwhile, professional identity should be integrated into one’s sense of self throughout professional education.

Second, our findings might help medical students better adapt to life events, particularly those with who have high expectations for finding work that is both meaningful and a satisfying fit with their identity. Academic institutions and medical educators could leverage these events to reshape medical students’ perceptions of work characteristics and occupational identity, and consequently discern their callings resulting from work sense-making and identity-formation. As job crafting can help people find deeper purpose and cultivate identity in work by altering task and relational boundaries [35], students are encouraged to use crafting techniques to find their “one true” calling created around work.

## Limitations and future research directions

The results of this study are promising, yet it has two limitations. First, given our cross-sectional study, researchers cannot make causal conclusions. It is highly likely that variations will be found in empirical studies involving medical students, future research could test the themes identified in our study to illuminate the emergence of calling. Secondly, although our sample aligned with our constructed definition, the convenience sampling procedure and sample size may reduce the transferability of results to individuals in other medical universities. A crucial next step is investigating a more diverse population of medical students.

## Data Availability

The datasets generated and/or analyzed during the current study are not publicly available as the knowledge maps generated as datasets allow conclusions to be drawn about the participants but are available from the corresponding author on reasonable request.
